# General immunity and superadditivity of two-way Gaussian quantum cryptography

**DOI:** 10.1038/srep22225

**Published:** 2016-03-01

**Authors:** Carlo Ottaviani, Stefano Pirandola

**Affiliations:** 1 Department of Computer Science & York Centre for Quantum Technologies, University of York, YO10 5GH, UK

## Abstract

We consider two-way continuous-variable quantum key distribution, studying its security against general eavesdropping strategies. Assuming the asymptotic limit of many signals exchanged, we prove that two-way Gaussian protocols are immune to coherent attacks. More precisely we show the general superadditivity of the two-way security thresholds, which are proven to be higher than the corresponding one-way counterparts in all cases. We perform the security analysis first reducing the general eavesdropping to a two-mode coherent Gaussian attack, and then showing that the superadditivity is achieved by exploiting the random on/off switching of the two-way quantum communication. This allows the parties to choose the appropriate communication instances to prepare the key, accordingly to the tomography of the quantum channel. The random opening and closing of the circuit represents, in fact, an additional degree of freedom allowing the parties to convert, a posteriori, the two-mode correlations of the eavesdropping into noise. The eavesdropper is assumed to have no access to the on/off switching and, indeed, cannot adapt her attack. We explicitly prove that this mechanism enhances the security performance, no matter if the eavesdropper performs collective or coherent attacks.

Quantum Key Distribution (QKD)^1^ is today one of the most advanced quantum technologies among those emerged from the fundamental research in quantum information. Rapidly progressing towards practical implementations^2^, the interest in QKD is motivated by the promise of achieving efficient distribution of cryptographic keys over insecure channels. In fact its main goal is to provide an information-theoretic secure strategy to share cryptographic keys in order to replace the current computationally-secure solution^3^, which has been proved to be vulnerable^4^ to attacks by quantum computers.

The typical scenario involves two parties, Alice and Bob, who want to share a secret message over an insecure channel^5^. To achieve this goal they encode classical information in non-orthogonal quantum states, which are sent over a noisy quantum channel under control of an eavesdropper, Eve. The standard assumptions to analyze the security of QKD protocols are the following: Eve is computationally unbounded, but has no-access to the parties’ private spaces^2^,^5^ and, most importantly, she is restricted by the no-cloning theorem^6^. The distribution of private keys is possible because any attempt to extract the encoded information unavoidably introduces noise on the quantum states. Monitoring this noise the parties can quantify how much Eve has learnt on the secret key and, consequently, apply classical error correction and privacy amplification protocols reducing Eve’s information to a negligible amount. Once they have distilled such a key, the parties can safely use the one-time pad protocol. In case the level of noise is too high, above the security threshold, they can to abort the protocol (denial of service).

The first theoretical proposals for QKD protocols have been designed for discrete variables (DV)^1^ systems. Today several remarkable implementation of DV-QKD have been achieved in both fibers^7^ and free space^8^. Beside this approach, several protocols exploiting quantum continuous-variable (CV) systems have been put forward^9^,^10^,^11^,^12^,^13^,^14^. In CV-QKD^15^ the information is encoded in quantum systems with continuous spectra (infinite-dimensional Hilbert space), and a special attention has been devoted to Gaussian CV systems^16^.

Gaussian CV-QKD has been achieved in *in-field* implementations^17^, with practical performances comparable to those of DV-QKD, despite the latter appears to be more robust for long-distances. The result of ref. 17 has been possible combining efficient reconciliation protocols^11^, post-selection^18^ techniques and efficient classical compression codes^19^. The interest in optical CV systems, for quantum information purposes, is now growing, boosted mainly by the natural properties of these systems: relatively cheap experimental implementation, higher rates, broadband detection techniques^20^, and the possibility of exploiting a wide range of frequencies^21^,^22^. These natural properties make CV-QKD a promising candidate for future practical real-world implementations, especially in the mid-range distances like the metropolitan areas where high rates are desirable^23^.

Today, many theoretical efforts are devoted to the design of device independent (DI) QKD protocols^24^,^25^. Despite recent remarkable results, the practical implementation of this approach remains still difficult^26^,^27^,^28^. Very likely the next generation of end-to-end quantum networks will use the recently introduced^29^,^30^ measurement device independent QKD (MDI-QKD) which allows the distribution of cryptographic keys preserving the protection against the most typical side-channel attacks, without the need to pass a Bell test (see ref. 29 for a general security analysis). Recently a very high-rate CV-MDI QKD protocol has been proposed and tested in a proof of principle experiment^23^,^31^,^32^.

Alongside the study of end-to-end QKD, it is also of great interest the design of more robust *point-to-point* QKD schemes improving the security performances of CV-QKD in noisier environments^14^ or able to exploit trusted noise^33^ to implement QKD at different frequencies^21^. In this regard, the two-way protocols^14^, where the parties make a double use of the quantum channel to improve the tolerance to noise, show higher security thresholds than the one-way counterparts. This idea has been also extended to thermal QKD^34^. Also note that the two-way protocols have been developed for DV-QKD^35^,^36^ and can be used for direct quantum communication^37^,^38^.

The main result in this work is the explicit study of the asymptotic security of two-way Gaussian protocols against coherent attacks, and the proof that these schemes are in fact immune to this eavesdropping. The general strategy to achieve this goal follows a previous insight^14^ and can be summarized as follows (see Fig. 1): The parties randomly switch ON or OFF the two-way communication line, and they post-select the OFF instances if they detect the presence of coherent attacks, otherwise they use the ON instances. We explicitly study the security threshold of the OFF configuration against two-mode coherent attacks, which are the residual eavesdropping after de Finetti reduction. Our approach allows us to prove that the superadditivity of the two-way thresholds is a general feature. This result can also be understood noting that the ON/OFF switching activates an additional degree of freedom, exclusive to the parties, which can be used to convert (a-posteriori) Eve’s correlations into a noise on which Eve has no control.

## Results

### The Scheme

**Figure 1:**
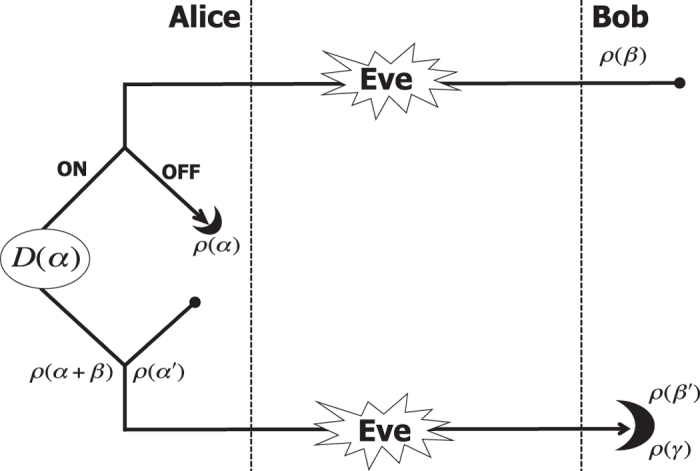
Two-way CV-QKD protocol. Steps: *forward*, Bob prepares coherent states of amplitude *β* and density matrix *ρ*(*β*) and sends them through the noisy channel. Using the circuit in ON configuration, Alice applies a random displacement 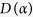 on 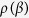 encoding information in the amplitude *α*. *Backward*, Alice then sends the quantum state 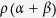 to Bob who applies heterodyne detection with outcome 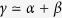, and applies classical post-processing to subtract the reference amplitude *β* to recover *α*. In OFF configuration, the circuit is opened at Alice’s station. She applies heterodyne detection on the reference state, obtaining the variable *α*. She then prepares a new coherent state 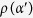 which is sent back to Bob who heterodynes this state obtaining the variable 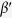. During the quantum communication Eve, as well as Bob, does not know which setup of the circuit has been adopted. For this reason, she is forced to attack both communication steps, and cannot adapt her attack to the ON/OFF setup.

In Fig. 1 we describe a two-way quantum communication protocol. We focus on use of coherent states, for the encoding, and heterodyne detections for the decoding^10^,^14^. Bob prepares a Gaussian-modulated reference coherent state with density matrix 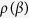, and use the quantum channel to transmit it to Alice who, randomly, decides to close (case ON) or open (case OFF) the quantum communication. Let discuss the two cases in detail.

*Case ON*: Alice encoding is performed by applying a Gaussian-modulated displacement 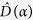 on the reference state 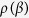, obtaining a new coherent state with density matrix 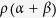. This is sent back to Bob who performs heterodyne detection on the received state 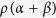, and applies classical post-processing in order to subtract the reference variable *β* and derive Bob’s estimate 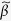 of Alice’s variable *α*.

*Case OFF*. Alice applies heterodyne detection on the reference state 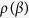 with outcome *α*. Then, she prepares a new Gaussian-modulated coherent state 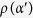 which is sent back to Bob, who heterodynes it with outcome 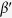. After this, the parties can use the two pairs of variables 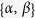 and 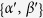 to prepare the key.

We note that, during the quantum communication, both Bob and Eve do not know the configuration adopted. This information is shared during the phase of parameter estimation and is part of the classical communication performed by Alice over the public channel. In the following we focus on the use of reverse reconciliation (RR)^11^,^16^ (direct reconciliation is discussed in the supplemental material). With the quantum communication in ON, the RR corresponds to Alice inferring Bob’s final outcome variable 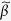. With the circuit in OFF, the RR corresponds to Bob estimating Alice’s variable *α* during the forward stage, followed by Alice estimating Bob’s detection variable 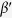.

As described in ref. 14 the advantage of having the ON/OFF switching is that this degree of freedom can be used to post-select the data in order to prepare the key. After the channel tomography they can determine which attack has been performed and in which status of the circuit. Then they keep data from case ON when they detect a collective attack, while they use data exchanged with the circuit in OFF when the attack is coherent.

### Security analysis and attack reduction

We study the security of the scheme assuming the asymptotic limit of many uses of the quantum channel, 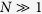. In the worst-case scenario the eavesdropper attaches ancillary quantum systems, 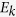, to each exchanged signal, and process the *E*_*k*_’s by a global coherent unitary operation. The ancillary output modes are stored in a quantum memory (QM), and coherently measured after the classical communication between Alice and Bob at the end of the protocol. Such an eavesdropping defines a general coherent attack.

The parties can now reduce the complexity of the previous scenario, by applying symmetric random permutations^39^ of their classical data. This allows them to get rid of all the correlations between distinct instances of the protocol. It is then clear that, in the case of two-way communication, the de Finetti symmetrization provides a residual two-mode coherent attack, where the only surviving correlations are those between 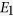 and 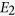, used by Eve in each single round-trip. These ancillary modes are mixed with the forward and backward signals by means of beam splitters. Note that we can rid of additional modes **e** because we work in the asymptotic limit and we bound Eve’s accessible information using the Holevo function^40^. Finally, a further simplification comes from the extremality of Gaussian states^16^, which means that we can restrict Eve’s input 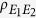 to be a Gaussian state.

**Figure 2:**
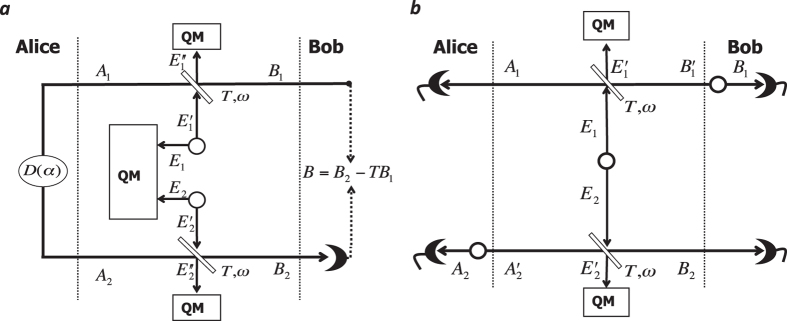
Panel (**a**) shows the two-way scheme in ON configuration. This is used against collective Gaussian attacks, typically implemented by means of two independent entangling cloners. Each beam-splitter has transmissivity *T*, and Eve mixes the ancillas 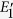 and 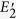 with the signals modes 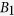 and 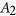 Panel (**b**) describes the OFF configuration, whose security is studied against two-mode Gaussian attacks. In this case we study the security of the scheme in the entanglement based representation.

The Gaussian attack is collective, when 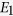, 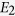 are uncorrelated, or two-mode coherent when they are correlated. Studying this second case with the circuit in ON and in DR, ref. 41 found that optimal two-mode attacks exist which can reduce the security performances of the two-way protocol below the one-way threshold. Here we show that, using the scheme with the ON/OFF switching, and post-selecting the optimal key-rate accordingly to the attack detected, the parties can overcome this problem. The security analysis is performed according to the setup shown in Fig. 2, where Fig. 2(a) refers to collective attacks, while Fig. 2(b) refers to two-mode coherent attacks. In the latter case, the security analysis is performed in the entanglement based (EB) representation^11^,^16^.

### Description of the two-mode Gaussian attack in the EB representation

In EB representation both Bob and Alice remotely prepare coherent states on the travelling modes 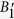 and 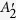 by using two-mode squeezed vacuum states, described by covariance matrices (CMs) of the following form


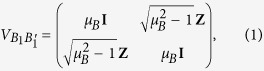



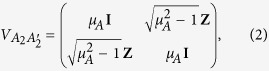


on which they apply heterodyne detections on the respective local modes 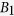 and 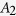. The parameters 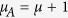, and 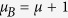 describe the variance of the thermal state injected by Alice and Bob, respectively. The two travelling modes, 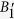 and 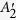, are mixed with Eve’s modes, 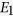 and 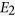, on two identical beam splitters, with transmissivity *T*. Eve’s input state 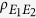 is a zero mean, two-mode correlated thermal state, with CM


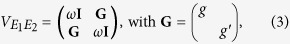


where 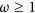 gives the variance of the thermal noise injected, while *g* and 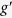 describe the correlations between the two ancillas.

Note that the double use of the channel corresponds to a sequential use of the same communication line (optical fibre), so it is reasonable to consider a symmetric channel (*T* and *ω* are the same during the forward and backward communication). The correlation parameters *g* and 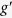 must fulfill the following constraints


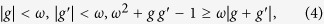


in order to certify that 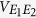 is a bona fide CM. If 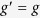, we must have 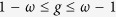, with the two extremal conditions corresponding to 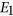 and 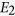 sharing maximal separable correlations^42^,^43^. If 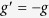 the ancillas share non-separable correlations. The Eq. (4) provides the bound 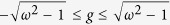, with the extremal values corresponding to maximally entangled states. Finally, if 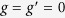, the two ancillas are not correlated, and the two-mode attack collapses to a standard collective one, based on two independent entangling cloners.

### Key-rates and security thresholds

We compute now the secret-key rate 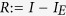, where *I* is Alice-Bob mutual information, and 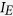 is Eve’s accessible information. In the asymptotic case 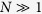, 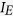 can be replaced by the Holevo information 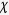. Hence we write


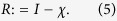


The goal of the security analysis is the computation of the bound 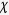, which is defined as


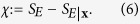


The functional 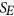 is the von Neumann entropy, relative to Eve’s quantum state 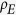, and 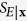 is that corresponding to 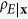, which describes Eve’s sate conditioned on the outcomes of the measurements performed by the parties.

Against collective attacks, the parties use the protocol in ON, and we have the following ON key-rate


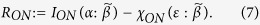


By contrast, against coherent attacks, they use the circuit in OFF, for which we have the following OFF key-rate


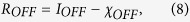


where


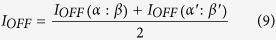


is the mutual information averaged over the forward and backward use, and


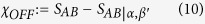


is computed on Alice and Bob’s output state *ρ*_*AB*_.

Thus, for collective attacks we select the ON key-rate 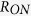, while for coherent attacks we select the OFF key-rate 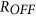. Both these key-rates are function of channel parameters, i.e., transmissivity *T* and excess noise 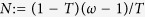 (which gives the extra noise on the channel with respect the vacuum shot-noise). The OFF key-rate, 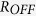, is also function of the correlation parameters *g* and 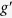. Therefore, once we have *R*, we find the security thresholds solving the following equation


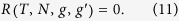


This condition provides threshold curves of the type 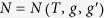 which simplifies to 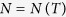 for collective attacks.

### Formulas for the key-rates

The computation of the secret-key rates can be performed using the mathematical tools described in ref. 16. From the knowledge of the CM describing the total and conditional states, we can compute the von Neumann entropies and finally the key rates. For the protocol with coherent states and heterodyne detection we find the following key-rates


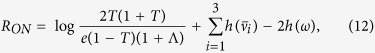



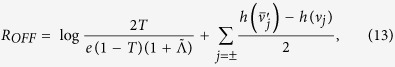


where


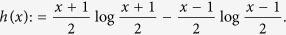


In the previous formulas, the symplectic eigenvalues 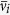 are computed numerically and we define 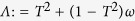 and 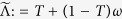. It is of particular interest the OFF key-rate of Eq. (13), from which we notice that one can recover the one-way key-rate in the case of collective attacks (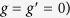. The expressions of the total and conditional symplectic eigenvalues can be computed analytically for large modulation, being equal to


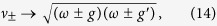



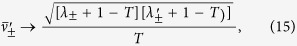


where 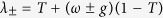 and 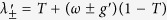.

### Protocol with coherent states and homodyne detection

Here we give the key-rate 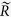 for the two-way protocol with coherent states and homodyne detection. The only change with respect to the previous scheme is clearly the use of homodyne detection for decoding. With the circuit in ON, Bob prepares coherent states, randomly displaced by Alice and finally homodyned by Bob. With the protocol in OFF, Bob prepares coherent states and Alice performs homodyne detection. Then she sends back newly prepared coherent states which are homodyned by Bob. After some algebra, we obtain the following analytical expressions for the key-rates


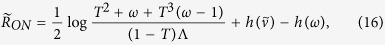



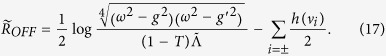


In the ON key-rate of Eq. (16), used against collective attacks, the asymptotic symplectic eigenvalue 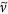 can be computed analytically as


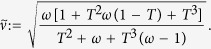


By contrast, the OFF key-rate of Eq. (17) is exploited under coherent attacks, and the total symplectic eigenvalues 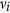 are the same as given in Eq. (14). The details to obtain Eqs. (16) and (17) can be found in the supplemental material, where we also include the secret-key rates computed in DR.

## Discussion

**Figure 3:**
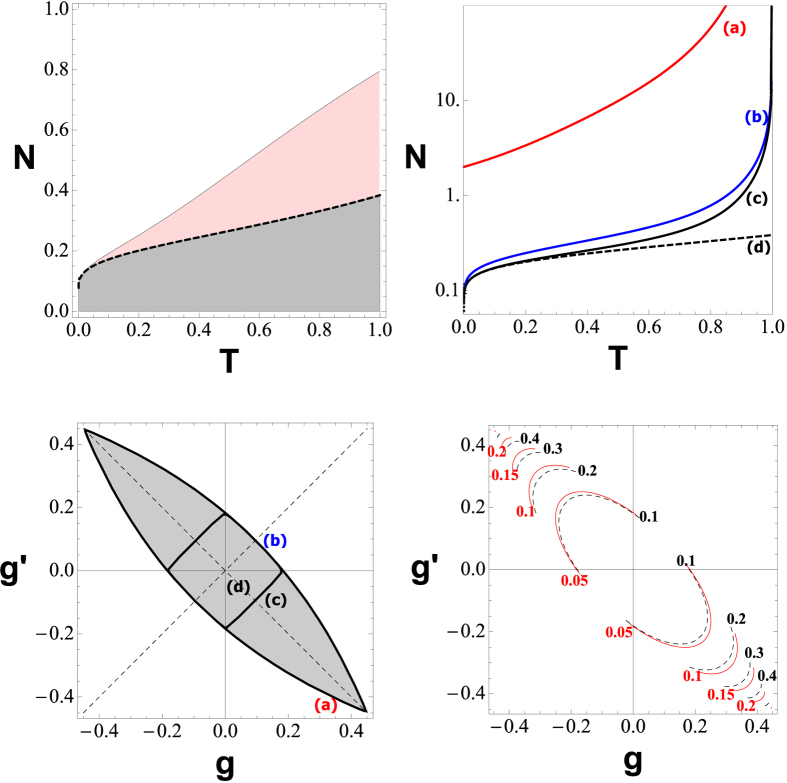
This figure summarizes the results for the protocol with coherent states and heterodyne detection, whose rates are given in Eqs. (12) and (13). The top panels describe the security thresholds, in terms of tolerable excess noise *N* versus transmissivity *T*. In the top-left panel, we consider collective attacks and we compare the ON two-way threshold 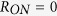 (black solid line) with the threshold of the one-way protocol (dashed line). In the pink region the two-way protocol is secure, while the one-way counterpart is not. In the top-right panel, we consider coherent attacks and we compare the OFF two-way threshold (**a**–**c**) with respect to the one-way threshold (**d**). In particular, curve (**a**) is obtained for 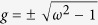, i.e., Eve using maximally entangled states; curve (**b**) considers the case 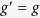 with 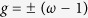; and curve (**c**) refers to 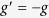 and 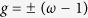. Note that curve (**d**) coincides with the OFF threshold against collective attacks, in which case the protocol is used in ON. The same labels (**a**–**d**) are used in the bottom-left panel, which describes the various attacks on the correlation plane 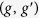, obtained setting 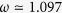 in the constraint of Eq. (4). Finally, in the bottom-right panel, we plot the OFF key-rate against coherent attacks (red lines), compared to the quantum mutual information (black lines) describing the correlations of Eve’s ancillas. We set 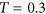 and 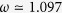, so that the one-way rate is 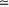 zero. We see that the OFF key-rate is always strictly positive and it increases for increasing correlations in the attack.

The security analysis of the thresholds coming from Eqs. (12) and (13) is summarized in Fig. 3. In particular, the security threshold for the ON configuration is confirmed^14^ to be superadditive in Fig. 3 (top-left). The black-solid line corresponds to the ON threshold, which is clearly above the threshold of the one-way protocol (dashed line). This comparison is done against collective attacks. The top-right panel of Fig. 3, shows the security threshold for the OFF configuration in the presence of several two-mode attacks with different values of the correlation parameters 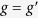. The curves labeled (a–d) corresponds to the points in Fig. 3 (bottom-left). For example, the red curve (a) describes coherent attacks performed with maximally entangled states. The curve (b) describes attacks with 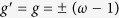, and the curve (c) corresponds to 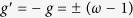. Finally, the dashed curve (d) corresponds to the center of the correlation plane, where the OFF threshold coincides with the one-way threshold. Thus, we see that for any value of Eve’s correlation parameters, *g* and 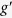, Alice and Bob can always post-select an instance of the two-way protocol (ON or OFF) whose threshold is strictly greater than that of the corresponding one-way protocol.

Finally, Fig. 3 (bottom-right) describes the connection between the OFF key rate and the amount of correlations in Eve’s ancillas, as quantified by the quantum mutual information. We can see that the OFF key rate not only is positive (while the one-way rate is always zero) but it also increases with Eve’s correlations, which are converted into noise by the OFF configuration. Thus, the ON/OFF switching, together with the post-selection of the correct instances, allows one to implement two-way CV-QKD in a way which is not only secure, but also more robust to excess noise with respect to one-way protocols under completely general attacks.

## Methods

A detailed description of the methods can be found in the supplementary material. The security analysis of the protocol has been performed in the entanglement based representation for the case OFF, so that we could compute the Holevo bound from the study of Alice-Bob CM. For the case ON in RR, we started from the output covariance matrix of Eve, to compute the total von Neumann entropy. We then computed the conditional von Neumann entropy completing Eve’s covariance matrix with the Bob’s mode on which we applied the heterodyne or the homodyne measurement, accordingly with the case studied.

## Conclusions

In this work we have studied the security of two-way CV-QKD addressing, explicitly, the superadditivity of its security threshold against coherent attack. To the best of our knowledge this represents the first attempt of such a complete study for direct point-to-point two-way protocols. Our security analysis is obtained assuming the asymptotic limit, i.e., large number of signals exchanged and large modulation. This allowed us to find closed formulas for the secret-key rates, from which we have proved that the two-way Gaussian protocols are more robust to excess noise than their one-way counterparts in both collective and coherent attacks.

For this property, it is crucial the random ON/OFF switching of the protocol, so that the eavesdropper’s correlations are under control of the parties and they are transformed, if needed, into useless noise. Our analysis contributes to the general understanding of the security properties of two-way protocols and is useful to extend CV-QKD to regime with high excess noise. Future developments could involve the study of this ON/OFF switching strategy in more complex quantum communication scenarios.

## Additional Information

**How to cite this article**: Ottaviani, C. and Pirandola, S. General immunity and superadditivity of two-way Gaussian quantum cryptography. *Sci. Rep.* **6**, 22225; doi: 10.1038/srep22225 (2016).

## Supplementary Material

Supplementary Information
