# Supplementary material for: General immunity and superadditivity of two-way Gaussian quantum cryptography
Source: Sci Rep. 2016 Mar 1;6:22225. doi: 10.1038/srep22225 (PMC4772010; doi:10.1038/srep22225)
Supplement: Supplementary Information [file srep22225-s1.pdf]

Supplemental Material for “General immunity and superadditivity of two-way Gaussian quantum cryptography”

Carlo Ottaviani and Stefano Pirandola

Department of Computer Science & York Center for Quantum Technologies, University of York, YO10 5GH, UK

ABSTRACT

This supplemental material gives the details of the calculations for the security analysis of the protocols described in this work, and includes also the key-rates calculated for two-way protocol used in direct reconciliation (DR), which has not been discussed in the main body. We underline that compared to the one-way protocol, the two-way presents a richer number of cases which need to be analyzed. The protocols are named with respect to the *preparation* and *detection* scheme adopted. Here we discuss the protocol with coherent states and heterodyne detection, and with coherent states and homodyne detection. Each one of previous cases can be implemented in DR as well as reverse reconciliation (RR), and here we give the results for both reconciliation schemes.

Secret-Key Rate and symplectic analysis

The secret-key rate quantifies the gap between Alice and Bob's mutual information and the information shared between Eve and the parties. Which parties' variable(s) has(have) to be considered depends on the setup of the protocol (one-way, two-way, ON or OFF) and, in general, from the reconciliation protocol employed.

For instance consider the one-way protocol. We assume that Alice sends a modulated coherent state with amplitude α to Bob, who receives a noisy version of this state, whose amplitude is β. The parties can then obtain two distinct secret-key rates defined as follows

$$R^{\blacktriangleright} := I(\alpha:\beta) - \chi(\varepsilon:\alpha), \tag{S1}$$
$$R^{\blacktriangleleft} := I(\alpha:\beta) - \chi(\varepsilon:\beta). \tag{S2}$$

The first describes the key-rate in DR, while the second the RR. The function I is the classical mutual information quantifying correlations between Alice's variable, α, and Bob's variable, β. For each quadrature measured, and used to encode information, the mutual information is given by the following general signal-to-noise ratio

$$I = \frac{1}{2}\log\frac{V}{V_C}, \tag{S3}$$

where V is the variance of the variable used to prepare the key, and V_C the conditioned variance of this statistical variable after the measurement performed by the parties.

In the asymptotic limit of many uses of the quantum channel we can bound Eve's accessible information by the Holevo function, which is given by

$$\chi(\varepsilon:x) := S(\varepsilon) - S(\varepsilon|x). \tag{S4}$$

The function $S(.)$ describes the von Neumann entropy which, for Gaussian quantum systems, has a simple form given by

$$S = \sum_k h(\nu_k), \tag{S5}$$

with the entropic function $h(.)$ is defined as follows

$$h(\nu_k) := \frac{\nu_k+1}{2}\log\frac{\nu_k+1}{2} - \frac{\nu_k-1}{2}\log\frac{\nu_k-1}{2}, \tag{S6}$$

and where the ν_k's are the symplectic eigenvalues of the CM which describes the dynamics of the studied Gaussian quantum system.[1]

The expression of the von Neumann entropy of Eq. (S6) can be further simplified exploiting the limit of large signal modulation, in which case we can write[1]

$$h(\nu_k) = \log \frac{e}{2}\nu_k + O\left(\frac{1}{\nu_k}\right). \tag{S7}$$

The computation of the symplectic spectra can be done in prepare and measure configuration, in which case the ν_k's are obtained from the symplectic analysis of Eve's output CM or, in case we use the equivalent EB representation, from Alice-Bob's CM. This second approach is used in the following, to study the OFF configurations, i.e., when we consider coherent attacks.

To compute the symplectic spectrum, we first compute the appropriate CM $\mathbf{V}$ and then, from matrix

$$\mathbf{M} = i\Omega\mathbf{V},$$

where $\Omega = \oplus_{i=1}^{n} \tilde{\omega}_i$, with $\tilde{\omega}_i$ the single-mode symplectic form given by

$$\tilde{\omega}_i = \begin{pmatrix} 0 & 1 \\ -1 & 0 \end{pmatrix},$$

we compute the ordinary eigenvalues, which come in pairs. The symplectic spectrum is obtained taking their absolute value.

Protocol with coherent states and heterodyne detection

We start showing how we obtain the ON key-rate for the protocol with coherent states and heterodyne detection, which is described in Fig. 2 (a) of the main text. The security analysis is performed using Eve's CM, obtained from the outputs $\{E_1, E_1'', E_2, E_2''\}$. From this we obtain the total von Neumann entropy and, by simple conditioning procedure, one can also compute Eve's conditional CM. This describes the conditional state $\rho_{E_1,E_1'',E_2,E_2''|\alpha}$, for the protocol in DR. By contrast, to study the protocol in RR, we complete Eve's output CM with Bob's post-processed output mode B, on which we apply the heterodyne detection in order to obtain the conditional CM for this case.

Case ON

Bob sends modulated coherent states to Alice providing, on average, a thermal state with variance $\mu_B = \mu + 1$, where μ accounts for the classical Gaussian modulation on the top of the vacuum shot-noise. Alice applies an additional random displacement, $D(\alpha)$, on the states received from Bob with modulation variance $\mu_{ON} = \mu \geq 0$.

Mutual information Alice-Bob mutual information can be computed from the expression of the variance of post-processed mode $\langle B^2 \rangle$, given by

$$\langle B^2 \rangle = [T^2 + T\mu_{ON} + (1 - T^2)\omega]I, \tag{S8}$$

from which, in the limit of large modulation $\mu_{ON} = \mu \to \infty$, we obtain the signal variance

$$V = T\mu. \tag{S9}$$

We then compute the conditional variance from Eq. (S8) by setting Alice modulation $\mu_{ON} = 0$, obtaining

$$V_C = T^2 + (1 - T^2)\omega. \tag{S10}$$

Finally, using Eqs. (S9) and (S10) with the expression of the mutual information in case of heterodyne detection

$$I := \log \frac{V + 1}{V_C + 1}, \tag{S11}$$

we obtain the following Alice-Bob mutual information in the limit of large modulation

$$I_{ON} = \log \frac{T\mu}{1 + T^2 + (1 - T^2)\omega}. \tag{S12}$$

Total Covariance Matrix We compute now the CM of Eve's output quantum state $\rho_{E_1',E_1'',E_2',E_2''}$. We arrange it in the following normal form

$$\mathbf{V}_E = \begin{pmatrix} \mathbf{A} & \mathbf{C} \\ \mathbf{C}^T & \mathbf{B} \end{pmatrix}, \tag{S13}$$

where

$$\begin{aligned} \mathbf{A} &:= \begin{pmatrix} \omega\mathbf{I} & \sqrt{T(\omega^2-1)}\mathbf{Z} \\ \sqrt{T(\omega^2-1)}\mathbf{Z} & \Psi\mathbf{I} \end{pmatrix}, \\ \mathbf{B} &:= \begin{pmatrix} \omega\mathbf{I} & \sqrt{T(\omega^2-1)}\mathbf{Z} \\ \sqrt{T(\omega^2-1)}\mathbf{Z} & \tilde{\Psi}\mathbf{I} \end{pmatrix}, \\ \mathbf{C} &:= \begin{pmatrix} 0 & \Xi\mathbf{Z} \\ 0 & \Phi\mathbf{Z} \end{pmatrix}, \end{aligned} \tag{S14}$$

with

$$\begin{aligned} \tilde{\Psi} &= [T\omega + (1-T)^2\omega + T(1-T)\mu_B] + (1-T)\mu_{ON}, \\ \Psi &= T(\omega-\mu_B) + \mu_B, \\ \Phi &= (1-T)\sqrt{T}(\mu_B-\omega), \\ \Xi &= -(1-T)\sqrt{(\omega^2-1)}. \end{aligned} \tag{S15}$$

From Eq. (S13) we easily obtain the total symplectic spectrum by taking the limits for $\mu_{ON} = \mu \to \infty$ and $\mu_B = \mu + 1 \to \infty$

$$\{\nu_1, \nu_2, \nu_3 \nu_4\} \to \{\omega, \omega, (1-T)^2\mu^2\}. \tag{S16}$$

The latter, used with Eqs. (S5) and (S7), gives the total von Neumann entropy

$$S_E = \log\frac{e^2}{4}(1-T)^2\mu^2 + 2h(\omega). \tag{S17}$$

Conditional CM and Key-rate in Direct Reconciliation For the DR the conditional CM can be obtained straightforwardly from Eq. (S13) setting $\mu_{ON} = 0$ on both quadratures in the block describing Eve's output E_2'', i.e., $\tilde{\Psi}$ in Eq. (S15). The resulting conditional CM has the following asymptotic symplectic spectrum

$$\{\bar{\nu}_1, \bar{\nu}_2, \bar{\nu}_3, \bar{\nu}_4\} \to \{1, 1, \omega, (1-T^2)\mu\}. \tag{S18}$$

Using Eq. (S18) with Eq. (S5) and (S7), we compute the conditional von Neumann entropy

$$S_{E|\alpha} = \log\frac{e}{2}(1-T^2)\mu + h(\omega). \tag{S19}$$

Now, using Eqs. (S17) and (S19) in Eq. (S4), one obtains the expression of the Holevo bound

$$\chi_{ON}^{\blacktriangleright} = \log\frac{e}{2}\frac{(1-T)}{(1+T)}\mu. \tag{S20}$$

Finally, by subtracting the Holevo function of Eq. (S20) from the mutual information of Eq. (S12) we get the ON key-rate in DR

$$R_{ON}^{\blacktriangleright} = \log\frac{2}{e}\frac{T(1+T)}{(1-T)[1+T^2+(1-T^2)\omega]} - h(\omega).$$

Reverse Reconciliation To study the security of the protocol in RR, we need to re-compute the conditional von Neumann entropy for this case. We complete the CM of Eq. (S13) adding the blocks describing Bob's output mode B and its correlations with the rest of Eve's modes. Then we apply a heterodyne detection on B obtain Eve's conditional CM after Bob's measurements. We then write

$$\mathbf{V}^{\blacktriangleleft} = \begin{pmatrix} \mathbf{V}_E & \bar{\mathbf{C}} \\ \bar{\mathbf{C}}^T & \bar{\mathbf{B}} \end{pmatrix}, \tag{S21}$$

where

$$\bar{\mathbf{B}}=[T^2+T\mu+(1-T^2)\omega]\mathbf{I},$$
$$\bar{\mathbf{C}}=\sqrt{1-T}\begin{pmatrix} \sqrt{T(\omega^2-1)}\mathbf{Z} \\ T(\omega-1)\mathbf{I} \\ \sqrt{(\omega^2-1)}\mathbf{Z} \\ \sqrt{T}[T(\omega-1)-\mu]\mathbf{I} \end{pmatrix}.$$

We then apply the formula for heterodyne detection[1] obtaining the following conditional CM

$$\mathbf{V}_C^{\blacktriangleleft}=\mathbf{V}_E+\bar{\mathbf{C}}(\bar{\mathbf{B}}+\mathbf{I})^{-1}\bar{\mathbf{C}}^T, \tag{S22}$$

which gives the following conditional symplectic spectrum

$$\{\bar{\nu}_1,\bar{\nu}_2,\bar{\nu}_3,\bar{\nu}_4\}\rightarrow\{\bar{\nu}_1,\bar{\nu}_2,\bar{\nu}_3,(1-T^2)\mu\}. \tag{S23}$$

Notice that the eigenvalues $\bar{\nu}_1,\bar{\nu}_2,\bar{\nu}_3$ are asymptotically depending only on the channel parameters (ω,T), and are related by the following expression

$$\bar{\nu}_1\bar{\nu}_2\bar{\nu}_3=\frac{[1+T^3+(1-T)(1+T^2)\omega]\omega}{T(1+T)}.$$

From the eigenvalues of Eq. (S23), used with Eqs. (S4), (S5) and (S7) we obtain the Holevo bound for the RR

$$\chi_{ON}^{\blacktriangleleft}=\log\frac{e}{2}(1-T^2)\mu+h(\bar{\nu}_1)+h(\bar{\nu}_2)+h(\bar{\nu}_3),$$

which, used with Eq. (S12) in the definition of Eq. (7)in the main text, gives the secret-key rate for the protocol used in RR given in Eq. (12) of the main text.

Case OFF

We now describe the details of the calculations for the protocol used in OFF, as described in Fig. 2 (b) of the main text. In this case we perform the security analysis considering two-mode coherent attacks, in the EB representation.

Total Covariance Matrix and von Neumann entropy

Bob starts from a two-mode squeezed vacuum state, described by the CM of Eq. (1) in the main text. Applying a local heterodyne detection on mode B_1, he projects the traveling mode B_1' in a coherent state. In the same way, Alice applies a local heterodyne detection on mode A_2, projecting the traveling mode A_2' in a coherent state. Finally, we assume that Eve injects the general Gaussian state described by Eq. (3) in the main text. Since the total state of Alice, Bob and Eve is pure, we can reduce ourselves to compute Alice and Bob's state (having the same entropy of Eve's). We then order Alice's and Bob's the output modes as follows $\{B_1, A_2, A_1, B_2\}$, and obtain the following expression

$$\mathbf{V}_{AB}^{OFF}=\begin{pmatrix} \tilde{\mathbf{A}} & \tilde{\mathbf{C}} \\ \tilde{\mathbf{C}}^T & \tilde{\mathbf{B}} \end{pmatrix}, \tag{S24}$$

where the matrix blocks have been defined as follows,

$$\tilde{\mathbf{A}}=\begin{pmatrix} \mu_B\mathbf{I} & & \tilde{\delta}\mathbf{I} \\ & \mu_A\mathbf{I} & \\ \tilde{\delta}\mathbf{I} & & \tilde{\tau}(\mu_B)\mathbf{I} \end{pmatrix},$$
$$\tilde{\mathbf{B}}=\tilde{\tau}(\mu_A)\mathbf{I},$$
$$\tilde{\mathbf{C}}=\begin{pmatrix} \mathbf{0} \\ \tilde{\delta}\mathbf{I} \\ (1-T)\mathbf{G} \end{pmatrix},$$

where

$$\mathbf{G}:=\begin{pmatrix} g & \\ & g' \end{pmatrix},$$

the coefficients $\tilde{\delta}$ and $\tilde{\tau}(y)$ have been defined as follows,

$$\tilde{\delta} := \sqrt{T[\mu_B{}^2 - 1]},$$
$$\tilde{\tau}(y) := (1-T)\omega + Ty. \tag{S25}$$

We compute the symplectic spectrum of CM (S24) and taking the asymptotic limit, $\mu_A = \mu_B \to \infty$, we obtain the following analytical expressions

$$\{\nu_\pm, \nu_3, \nu_4\} \to \{\sqrt{(\omega \pm g)(\omega \pm g')}, (1-T)\mu, (1-T)\mu\}, \tag{S26}$$

which gives the total von Neumann entropy for the case OFF

$$S_{AB} = \log\left(\frac{e}{2}\right)^2 (1-T)^2\mu^2 + h(\nu_-) + h(\nu_+). \tag{S27}$$

Conditional covariance matrix and Alice-Bob mutual information

To obtain the conditional CM in DR we set $\mu_A = \mu_B = 1$, in modes B_1 and A_2. It is easy to verify that the resulting CM has the following symplectic spectrum

$$\{\bar{\nu}_1, \bar{\nu}_2, \bar{\nu}_+, \bar{\nu}_-\} \to \{1, 1, \sqrt{\lambda_+\lambda'_+}, \sqrt{\lambda_-\lambda'_-}\}, \tag{S28}$$

where, $\lambda_\pm = T + (\omega \pm g)(1-T)$ and $\lambda'_\pm = T + (\omega \pm g')(1-T)$. Using these eigenvalues, we compute the following conditional von Neumann entropy

$$S_{AB|\alpha',\beta} = h(\bar{\nu}_+) + h(\bar{\nu}_-).$$

The previous equation and Eq. (S27) are then used to obtain the asymptotic expression of the Holevo function in DR, which is given by

$$\chi^{\blacktriangleright}_{OFF} := S_{AB} - S_{AB|\alpha,\beta'}. \tag{S29}$$
$$= \log\frac{e}{2}(1-T)^2\mu^2 + \frac{1}{2}\sum_{k=\pm}[h(\nu_k) - h(\bar{\nu}_k)]. \tag{S30}$$

The conditional CM corresponding to the RR, is obtained by applying two consecutive heterodyne detections, starting from CM of Eq. (S24). We first measure mode B_2 and then we apply another heterodyne detection on mode A_2 (the order of these two local measurements is of course irrelevant). The resulting conditional CM has the symplectic spectrum

$$\bar{\nu}'_\pm \to \frac{\sqrt{[\lambda_\pm + 1 - T][\lambda'_\pm + 1 - T)]}}{T}.$$

These are used to compute the Holevo bound. We find Eq. (S29)

$$\chi^{\blacktriangleleft}_{OFF} = \log\frac{e}{2}(1-T)^2\mu^2 + \frac{1}{2}\sum_{k=\pm}[h(\nu_k) - h(\bar{\nu}'_k). \tag{S31}$$

Alice-Bob Mutual Information and Secret-key rate.

Alice-Bob mutual information is easily computed from the coefficient $\tilde{\tau}$, given in Eq. (S25). Taking the limit of large modulation, using the formula defining the mutual information for heterodyne detections (S11), and averaging over the double use of the quantum channel we obtain the following expression

$$I_{OFF} = \log\frac{T\mu}{1+\tilde{\Lambda}}, \tag{S32}$$

where $\tilde{\Lambda} = T + (1-T)\omega$. Notice that, differently from the Holevo bound, the mutual information is independent from the correlation parameters described by the matrix $\mathbf{G}$.

Using Eq. (S30) and Eq. (S32), and after some simple algebra, we get the analytical expression of the key-rate in DR

$$R^{\blacktriangleright}_{OFF} = \log\frac{2T}{e(1-T)(1+\tilde{\Lambda})} + \frac{1}{2}\sum_{k=\pm}[h(\bar{\nu}_k) - h(\nu_k)], \tag{S33}$$

The key-rate in RR of Eq. (13)in the main text, is obtained using previous Eq. (S31) and Eq. (S32).

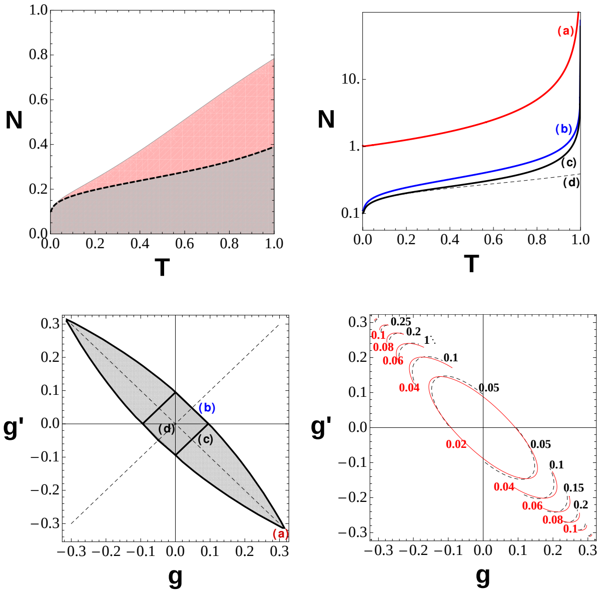

Figure S1. This figure summarize the results for the protocol with coherent states and homodyne detection, whose rates are given in Eqs. (S39) and (S46) which are the same of Eqs. (16) and (17) in the main text. The top panels give the security thresholds in terms of tollerable excess noise N versus transmissivity T. In the top-left panel, we consider collective attacks and we compare the ON two-way threshold $\tilde{R}_{ON} = 0$ (black solid line) with the threshold of the one-way protocol (dashed line). In the pink region the two-way protocol is secure, while the one-way counterpart is not. In the top-right panel, we consider coherent attacks and we compare the OFF two-way threshold (a)-(c) with respect to the one-way threshold (d). In particular, curve (a) is obtained for $g = \pm\sqrt{\omega^2 - 1}$, i.e., Eve using maximally entangled states; curve (b) considers the case $g' = g$ with $g = \pm(\omega - 1)$; and curve (c) refers to $g' = -g$ and $g = \pm(\omega - 1)$. Note that curve (d) coincides with the OFF threshold against collective attacks, in which case the protocol is used in ON. The same labels (a)-(d) are used in the bottom-left panel, which describes the various attacks on the correlation plane (g, g'), obtained setting $\omega \simeq 1.049$ in the constraint of Eq. (4) in the main text. Finally, in the bottom-right panel, we plot the OFF key-rate against coherent attacks (red lines), compared to the quantum mutual information (black lines) describing the correlations of Eve's ancillas. We set $T = 0.2$ and $\omega \simeq 1.049$, so that the one-way rate is $\simeq$ zero. We see that the OFF key-rate is always strictly positive and it increases for increasing correlations in the attack.

Protocol with coherent states and homodyne detection

In contrast to the protocol analysed in previous section, here the decodings are performed by means of homodyne detections. This modifies the expression of the mutual information and these of the conditional von Neumann entropies.

Case ON

Direct Reconciliation: conditional covariance matrix The conditional CM in DR is obtained as before. We start from the total CM of Eq. (S13) and we apply the following conditioning procedure

$$\bar{\mu}^q_{ON} = 1/\mu \overset{\mu\to\infty}{\to} 0, \qquad \bar{\mu}^p_{ON} = \mu, \tag{S34}$$

which describes Alice's effective modulation in order to describe the measurement of only one quadrature during the decoding stage (homodyne detection).

Direct Reconciliation: mutual information, Holevo bound and key-rate The conditioning procedure, described by Eqs. (S34), can clearly also used to determine Alice-Bob mutual information. In the present case only one-quadrature is used to encode the key so Alice-Bob mutual information is given by the following expression

$$\tilde{I}_{ON} = \frac{1}{2} \log \frac{T\mu}{T^2 + (1 - T^2)\omega}. \tag{S35}$$

Eve's conditional CM is obtained from Eq. (S13) applying recipe of Eqs. (S34). One easily obtains the conditional symplectic spectrum which, in the asymptotic limit, is given by

$$\{\nu_1^{\blacktriangleright}, \nu_2^{\blacktriangleright}, \nu_3^{\blacktriangleright}\nu_4^{\blacktriangleright}\} \to \{1, \omega, \sqrt{(1-T)^2(1-T^2)\omega\mu^3}\}. \tag{S36}$$

After some algebra we obtain the following Holevo bound

$$\tilde{\chi}_{ON}^{\blacktriangleright} = \frac{1}{2}\log\frac{(1-T)^2\mu}{(1-T^2)\omega} + h(\omega),$$

which, used with Eq. (S35), provides the ON key-rate for the protocol in DR

$$\tilde{R}_{ON}^{\blacktriangleright} = \frac{1}{2}\log\frac{T(1+T)\omega}{(1-T)[T^2+(1-T^2)\omega]} - h(\omega). \tag{S37}$$

It is interesting to note that plotting the security threshold of the key-rate of Eq. (S37), one finds that it provides a positive key-rate even below 3 dB, which sets the limit performance of the one-way version of this protocol in DR.

Reverse Reconciliation: conditional covariance matrix The security of the protocol is performed repeating the steps described in previous sections, replacing the heterodyne detections with homodyne measurements on B. Indeed, we apply the following formula

$$\tilde{\mathbf{V}}_C^{\blacktriangleleft} = \mathbf{A} - \mathbf{C}(\Pi\bar{\mathbf{B}}\Pi)\mathbf{C}^T,$$

to Eq. (S21). Note that $\Pi :=\mathrm{diag}(1,0)$ $(\mathrm{diag}(0,1))$ for heterodyne on quadrature $\hat{q}$ $(\hat{p})$. We then compute the symplectic spectrum of CM $\tilde{\mathbf{V}}_C^{\blacktriangleleft}$, which we rewrite here in the following form

$$\begin{aligned} \tilde{\nu} &\to \sqrt{\frac{\omega[1+T^2\omega - T^3(\omega-1)]}{T^2+\omega+T^3(\omega-1)}}, \\ \nu_2^{\blacktriangleleft} &\to \omega, \\ \nu_3^{\blacktriangleleft}\nu_4^{\blacktriangleleft} &\to \sqrt{\frac{(1-T)^3[T^2+\omega+T^3(\omega-1)]\mu^3}{T}}. \end{aligned} \tag{S38}$$

We then obtain the Holevo bound $\tilde{\chi}_{ON}^{\blacktriangleleft}$

$$\tilde{\chi}_{ON}^{\blacktriangleleft} = h(\omega) - h(\tilde{\nu}) + \frac{1}{2}\log\frac{T(1-T)\mu}{T^2+\omega+T^3(\omega-1)},$$

which combined with the mutual information of Eq. (S35) gives the ON key-rate in RR of Eq. (16) of the main text, i.e.,

$$\tilde{R}_{ON}^{\blacktriangleleft} = \frac{1}{2}\log\frac{T^2+\omega+T^3(\omega-1)}{(1-T)\Lambda} + h(\tilde{\nu}) - h(\omega), \tag{S39}$$

with $\Lambda := T^2 + (1-T^2)\omega$.

Case OFF

This case is studied in the EB representation, following the same steps of the previous OFF case, for both the DR and RR, replacing the final heterodyne with homodyne detections. The mutual information is computed averaging over the double use of the quantum channel, i.e., using the following definition of the mutual information

$$\tilde{I}_{OFF} := \frac{1}{2}\left(\frac{1}{2}\log\frac{T\mu_A}{(1-T)\omega+T\mu_A} + \frac{1}{2}\log\frac{T\mu_B}{(1-T)\omega+T\mu_B}\right) \overset{\mu_A=\mu_B=\mu\to\infty}{=} \frac{1}{2}\log\frac{T\mu}{\tilde{\Lambda}}, \tag{S40}$$

where $\tilde{\Lambda} := T + (1-T)\omega$.

Direct Reconciliation

The steps to compute the conditional CM have been discussed previously, so here we just provide the analytical expressions of the conditional symplectic spectra

$$\tilde{\nu}_{\pm}^{\blacktriangleright} = \sqrt{(1-T)\Gamma_{\pm}\mu}, \tag{S41}$$

$$\tilde{\eta}_{\pm}^{\blacktriangleright} = \sqrt{\frac{(\omega \pm g')[T+(\omega \pm g)(1-T)]}{\Gamma_{\pm}}}, \tag{S42}$$

where we define

$$\Gamma_{\pm} := 1-T+T(\omega \pm g'). \tag{S43}$$

Notice that, in previous spectra, the role of the correlation parameters depends on the quadrature measured by the homodyne detection of the decoding stage. We obtain the following Holevo bound for the DR

$$\tilde{\chi}_{OFF}^{\blacktriangleright} = \frac{1}{2}\log\frac{(1-T)\mu}{\sqrt{[1+t(\omega-1)]^2-T^2g^2}},$$

and the key-rate in direct reconciliation is given by

$$\tilde{R}_{OFF}^{\blacktriangleright} = \frac{1}{2}\log\frac{T\sqrt{[1+T(\omega-1)]^2-T^2g^2}}{(1-T)[T+(1-T)\omega]} - \sum_{k=\pm}\frac{h(\tilde{\eta}_k^{\blacktriangleright})-h(\nu_k)}{2}, \tag{S44}$$

where the eigenvalues $\nu_{\pm}$ are defined in Eq. (S26).

Reverse Reconciliation

For the RR, when the homodyne detection is performed on the quadrature $\hat{q}$, we obtain the following conditional symplectic eigenvalues

$$\tilde{\nu}_{\pm}^{\blacktriangleleft} = \sqrt{\frac{(1-T)(\omega \pm g)\mu}{T}}. \tag{S45}$$

By contrast, in case of homodyne detection on $\hat{p}$, the corresponding eigenvalues can be obtained from Eq. (S45) by exchanging $g \longleftrightarrow g'$. Averaging over the two detections, we find the following Holevo bound

$$\tilde{\chi}_{OFF}^{\blacktriangleleft} = \sum_{k=\pm}\frac{h(\nu_k)}{2} + \frac{1}{2}\log\frac{T(1-T)\mu}{\sqrt[4]{(\omega^2-g'^2)(\omega^2-g^2)}},$$

which subtracted to the mutual information of Eq. (S40) gives the key-rate of Eq. (17) of the main text, i.e.,

$$\tilde{R}_{OFF}^{\blacktriangleleft} = \frac{1}{2}\log\frac{\sqrt[4]{(\omega^2-g^2)(\omega^2-g'^2)}}{(1-T)\tilde{\Lambda}} - \sum_{k=\pm}\frac{h(\nu_k)}{2}. \tag{S46}$$

References

1. Weedbrook, C., et al. Gaussian quantum information, Rev. Mod. Phys. **84**, 621 (2012).
